# A Rare Case of Bilateral Tubal Ectopic Pregnancy Following Intracytoplasmic Sperm Injection-Embryo Transfer (ICSI-ET)

**DOI:** 10.1055/s-0040-1708093

**Published:** 2020-03

**Authors:** Ferruh Acet, Ege Nazan Tavmergen Goker, Ismet Hortu, Gulnaz Sahin, Erol Tavmergen

**Affiliations:** 1 Department of Obstetrics and Gynecology, Ege University Faculty of Medicine, Izmir, Turkey

**Keywords:** bilateral tubal ectopic pregnancy, in vitro fertilization, assisted reproductive technology, methotrexate

## Abstract

Bilateral tubal ectopic pregnancy is a very rare form of ectopic pregnancy. The incidence is higher in women undergoing assisted reproductive techniques or ovulation induction. We report the case of bilateral tubal ectopic pregnancy. The patient was 30 years old and had a 3-year history of infertility; she was referred to the in-vitro fertilization (IVF) program because of tubal factor infertility. A pregnancy resulted from the transfer of two embryos during an artificial cycle. Despite the increase in β-hCG values during the follow-up, 22 days after the embryo transfer, the β-hCG levels were 2,408 U/L and the serum progesterone (P4) level was 10.53 ng/ml. After application with methotrexate, β-hCG levels did not decrease effectively. Moreover, the sonographic screening revealed a suspicious bilateral tubal focus for ectopic pregnancy. A mini-laparotomy was performed and a bilateral tubal pregnancy was found. In the case of unilateral tubal pregnancy after the transfer of two embryos, the situation of the other tube should be systematically checked and β-hCG levels should be monitored.

## Introduction

Ectopic pregnancies (EPs) are known to occur with increased frequency after in-vitro fertilization (IVF) and related techniques. The incidence of EP as a result of assisted reproductive technology (ART) is 1.4 to 5.4%.^1^ Bilateral tubal pregnancies are extremely rare in spontaneous pregnancies, with an incidence of only 1 in 200,000. The reported rate of bilateral tubal pregnancies after ART is 1 in every 750 to 1,850 ectopic pregnancies.^2^ Misdiagnosis or delayed diagnosis of EP leads to such complications as severe bleeding and hypovolemic shock associated with maternal morbidity and mortality. Pelvic inflammatory disease, tubal infertility, previous pelvic surgery, and congenital uterine abnormalities have been defined as the leading factors contributing to the risk of EP in women with infertility.^3^ ^4^ ^5^ The number and quality of transferred embryos, controlled ovarian stimulation (COS) regimens, laboratory conditions, and transfer techniques have been offered as possible contributors to the occurrence of EP after IVF-embryo transfer (ET).^6^ Accurate management strategies for patients at risk of ectopic gestation after IVF-ET procedure has not been established. This case report deals with this rare condition, bilateral tubal pregnancy, seen in association with IVF-ET. The case of a 30-year-old patient with bilateral tubal ectopic pregnancy is presented.

## Case Report

**Figure 1:**
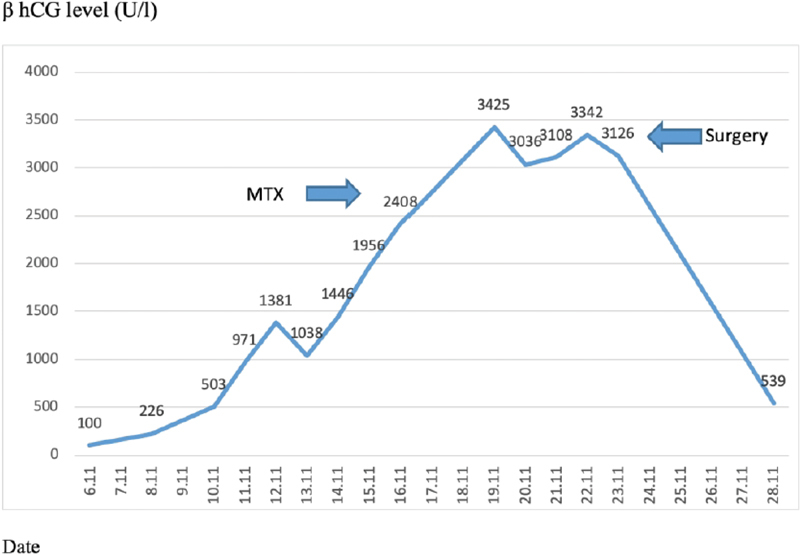
Levels of β-hCG in the follow-up period.

A 30-year-old woman with a 3-year history of primary infertility was referred to the IVF program. Her medical history revealed a laparoscopic surgery due to suspicion of fallopian tube pathology on hysterosalpingography (HSG). No tubal passage was found on HSG. Her basal hormone profile was found to be within the normal limits, and the transvaginal ultrasonography (TVUSG) revealed bilateral polycystic ovaries and normal uterus on the 3^rd^ day of the menstrual cycle. Antagonist protocol and recombinant follicle-stimulating hormone and human menopausal gonadotropin starting from day 3 of the menstrual cycle were used for ovulation induction. Follicular development was monitored by plasma estradiol (E2) and TVUSG. On the 10^th^ day of the cycle, with follicles over 18 mm and estradiol levels of 3,995 ng/ml, 250 µcg of human chorionic gonadotropin (hCG) was administered. Transvaginal oocyte retrieval was performed 36 hours after hCG, yielding 21 oocytes. To prevent ovarian hyperstimulation syndrome the freeze-all strategy was used, and all embryos were frozen. The patient underwent a frozen embryo transfer (FET) cycle 10 months later and failed to conceive. A pregnancy resulted from the transfer of two embryos during an artificial cycle in her second FET cycle. Fourteen days after the transfer of the embryos, the β-hCG level was 100 U/L. Serial monitoring of serum β-hCG values are shown in Fig. 1. The first values of β-hCG were obtained from a regular increase pattern for an intrauterine pregnancy. On the 17th day of transfer, the patient was referred to our clinic due to a decrease and a following increase in β-hCG, after which she was hospitalized.

**Figure 2:**
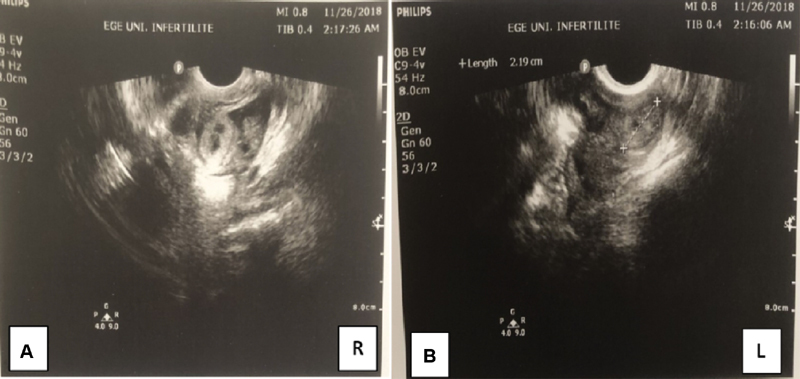
(**a, b**) Hematosalpinx on both tubes with suspicion of ectopic pregnancy and free fluid in the pouch of Douglas.

**Figure 3:**
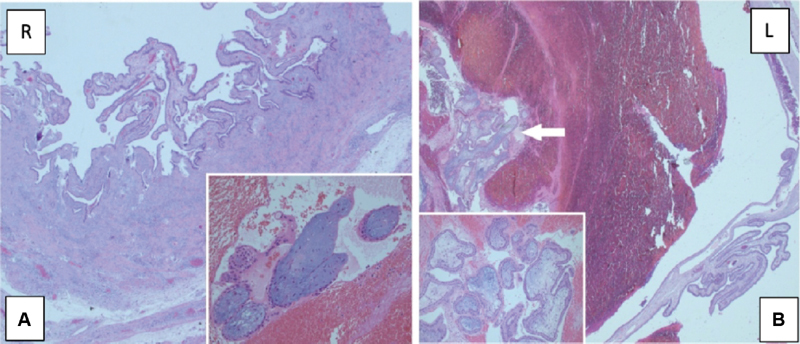
**(a, b)** Right fallopian tube: Edema and decidualized stromal areas in the perforated tubal wall (H&E 25x). Degenerated villi structures in the blood-fibrin mass falling from the perforated area of the fallopian tube (H&E 200x) **b.** Left fallopian tube: The villi structures between the blood-fibrin masses (shown by the arrow) in the lumen of the fallopian tube (H&E 25x). Magnification of villus structures (H&E 200x).

Despite the increase in β-hCG values during the hospital follow-up, 22 days after the embryo transfer, the β-hCG levels were 2,408 U/L and the serum progesterone (P4) level was 10.53 ng/ml. The ultrasound revealed a 12 × 15 mm gestational sac in the left fallopian tube, the endometrial thickness was 10.7 mm, and an ectopic pregnancy was diagnosed. A single dose of methotrexate (MTX, 50 mg/m2) was administered. On the 4th day of the treatment, β-hCG level was 3,036 U/L, and on the 7th day, it was 3,122 U/L. After treatment with methotrexate, β-hCG levels started increasing again after some decrease. The patient developed pelvic pain and vaginal bleeding. The transvaginal ultrasonography revealed hematosalpinx on both tubes, with a suspicion of a gestational sac in the right fallopian tube, and free fluid in the pouch of Douglas (Fig. 2 a,b). Due to severe adhesions on her previous operation report, a mini laparotomy was performed, which confirmed the diagnosis of bilateral tubal ectopic pregnancy. The right fallopian tube was ruptured from the ampullary portion, the left fallopian tube was intact but distended with blood and ∼ 300 ml free blood was evacuated from the pelvis. Bilateral salpingectomy was performed with pathologic confirmation of diagnosis chorionic villi identified in both tubes (Fig. 3 a,b). The microscopical examination of the materials revealed decidua but no chorionic villi. The patient had an uneventful recovery. The levels of β-hCG had returned to below 50 U/l at the 3^rd^postoperative week.

## Discussion

Ectopic pregnancy occurs in ∼ 2% of all clinical pregnancies in ART cycles, and it is an extremely rare condition. Tubal pathology is the main risk factor for ectopic pregnancy, after IVF.^7^ Most of the case reports that have been described of bilateral EP after ART in women presented known tubal disease. Uni- or bilateral tubal pathology, previous pelvic surgery, and pelvic infections are associated with EP. Recent studies have showed that pelvic inflammatory disease and subsequent tubal occlusion have been described as risk factors for EP following both natural and assisted conception.^8^ ^9^ Deep fundal or rapid embryo transfer in tubal factor infertility patients may result in direct injection of transfer media into a dysfunctional fallopian tube. Poor embryo quality, as well as the use of multiple embryo transfer, is a risk factor for extrauterine implantation. There are also reports suggesting that gonadotropin-releasing hormone (GnRH) analogue use may be linked to a higher rate of EP in the IVF population.^10^ In this case, infertility due to tubal damage and severe pelvic adhesions were the most important risk factors. Blastocyst and frozen embryo transfer might be beneficial for minimizing the risk of EP. It has been proposed that the volume of the transferred medium should not exceed 20 μL, according to the “spray effect” theory.^11^ The most reliable technique for the diagnosis of EP is TVUSG. The absence of an intrauterine gestational sac with rising β-hCG level is consistent with an abnormal pregnancy. Serial β-hCG levels can be also be used for diagnosis. The minimal rise in β-hCG for a viable intrauterine pregnancy is 53% in 2 days. The minimal decline in a spontaneous abortion is 21 to 35% in 2 days, depending on the initial level. A rise or fall in serial β-hCG values lower than these values, is suggestive of an ectopic pregnancy.^5^

In the present case, an initially rapid rise in β-hCG levels was observed, probably caused by the two sources (both fallopian tubes) of hormone production. This also led to the misinterpretation that this was an intrauterine pregnancy. A combination of sonography, pelvic examination and follow-up of the serum β-hCG levels was the crucial key element behind adequate management of the current case. Close follow-up via ultrasonography and physical examination helped us with early diagnosing and surgical management of the present case. Moreover, bilateral tubal trophoblastic activity resulting in higher β-hCG than a unilateral tubal ectopic pregnancy can confuse the clinician's decisions. Delay in diagnosing tubal ectopic pregnancy might lead to severe intraabdominal bleeding and even maternal death. However, as in our case, accurate follow-up by expert physicians enabled us to manage successfully both patient and clinician early .

Methotrexate is a folic acid antagonist that inactivates de novo synthesis of cellular DNA. This antineoplastic, antimetabolic drug has been increasingly used to treat EP since 1982.^12^ To date, therapeutic options for EP include surgery, medical treatment, or expectant management. Systemic MTX treatment has been accepted as a cost-effective alternative to laparoscopy for hemodynamically stable patients. Intermittent β-hCG measurements have been used for the diagnosis of EP and for the evaluation of the efficacy of MTX. As discussed previously, because of two sources of β-Hcg, the course of hormone may result in incorrect evaluation. Recent reports show that in bilateral EP patients, the contralateral tubal pregnancy was diagnosed from days to weeks after the initial surgery, so clinicians must be aware of such an alternative, especially in tubal infertility patients in whom multiple embryo transfers have been performed, the contralateral fallopian tube should always be examined and inconsistent β-hCG values may cause delay of accurate diagnosis.

## Conclusion

In conclusion, it should be noted that no specific diagnostic criteria exist for bilateral tubal ectopic pregnancy. This paper highlights to clinicians the importance of bilateral ectopic pregnancies after IVF. Taken together, close follow-up and sonographical screening, of both the uterine cavity and the adnexal region, should be recommended.
